# Perioperative immunotherapy for advanced resectable melanoma: a cost-effectiveness analysis

**DOI:** 10.1186/s12913-026-14258-y

**Published:** 2026-02-26

**Authors:** Mingjun Rui, Qiran Wei, Yingcheng Wang, Joyce H.S. You

**Affiliations:** School of Pharmacy, Faculty of Medicine, The Chinese University of Hong Kong, Hong Kong SAR, China

**Keywords:** Perioperative, Immunotherapy, Melanoma, Cost-effectiveness analysis

## Abstract

**Background:**

Recent studies have shown the efficacy of perioperative immunotherapy in improving the event-free survival of patients with advanced resectable melanoma. This study evaluated the cost-effectiveness of different perioperative immunotherapy treatment strategies versus adjuvant immunotherapy treatment strategies from the US payer perspective.

**Methods:**

Two immunotherapy strategies were each compared with the corresponding standard adjuvant treatment in two Markov models: Model (1) perioperative nivolumab plus ipilimumab versus adjuvant nivolumab for stage III patients; model (2) perioperative pembrolizumab versus adjuvant pembrolizumab for stage IIIB to IVC patients. Primary outcomes included direct costs, life-year gained (LYG), quality-adjusted life-year (QALY), and incremental cost per QALY (ICER). Sensitivity analyses were conducted to evaluate the robustness of the model outcomes.

**Results:**

In base-case analysis, perioperative nivolumab plus ipilimumab (versus adjuvant nivolumab) gained higher QALY (by 2.73) with cost-saving (by USD109,157) in model (1), and perioperative pembrolizumab (versus adjuvant pembrolizumab) gained higher QALY (by 2.29) with cost-saving (by USD130,157) in model (2). Both perioperative strategies were accepted as cost-effective. One-way sensitivity analyses found the base-case results robust to variation in all model parameters at willingness-to-pay threshold of 100,000 USD/QALY. Probabilistic sensitivity analysis demonstrated that the two perioperative strategies were accepted as cost-effective in 100% of 10,000 simulations.

**Conclusions:**

Perioperative immunotherapies demonstrated cost-saving compared with adjuvant immunotherapies from the US payer perspective. One-way and probabilistic sensitivity analyses supported the robustness of the cost-effectiveness results.

**Clinical trial number:**

Not applicable.

**Supplementary Information:**

The online version contains supplementary material available at 10.1186/s12913-026-14258-y.

## Background

Melanoma is a kind of skin cancer that starts in the melanocytes. While stage II-IV melanoma is often treated with surgical resection, the risk of relapse ranges between 30% and 90% after complete resection [1, 2]. Globally, the incidence of melanoma has been rising, particularly among fair-skinned populations in high-income countries such as the United States [3]. The current standard of care for patients with resectable melanoma is complete surgical excision of the primary tumor and involved lymph nodes, plus adjuvant systemic therapy [4, 5]. Adjuvant systemic therapy includes immune checkpoint inhibitors (ICIs) such as anti-programmed cell death protein 1(PD-1) agents (nivolumab and pembrolizumab) and targeted therapy for B-Raf Proto-Oncogene, Serine/Threonine Kinase (BRAF)-mutant tumors (dabrafenib plus trametinib) [4, 6]. 

Recent studies have explored the role of immunotherapy in the perioperative setting for patients with resectable melanoma [7]. NADINA trial, a multinational phase 3 study conducted in Europe and Australia (*n* = 423), showed that neoadjuvant ipilimumab plus nivolumab (followed by surgery and response-adapted adjuvant therapy) significantly improved event free survival (EFS) when compared with surgery followed by standard adjuvant therapy in patients with resectable stage III melanoma, with a hazard ratio (HR) for progression recurrence or death of 0.32 (99.9% CI 0.15 to 0.66) [8]. In the SWOG S1801 trial (*n* = 313) conducted in the US, patients with stage IIIB to IVC melanoma who received neoadjuvant pembrolizumab followed by surgery and continued adjuvant therapy had significantly improved EFS when compared with surgery followed by adjuvant pembrolizumab alone, with a HR of 0.58 (95% CI 0.39 to 0.87) [9]. These findings supported the consideration of perioperative immunotherapy as a component of clinical management for patients with advanced melanoma.

The perioperative immunotherapy strategies with nivolumab plus ipilimumab and with pembrolizumab are associated with improved EFS, but the cost-effectiveness is yet to be investigated. To provide health economics evidence to assist the decision-making process of clinicians and healthcare payers, this study assessed the cost-effectiveness of two perioperative treatment strategies (nivolumab plus ipilimumab, and pembrolizumab) versus the current standard of care (adjuvant therapy) in patients with resectable melanoma from the US payer perspective.

## Methods

### Model design

**Fig. 1:**
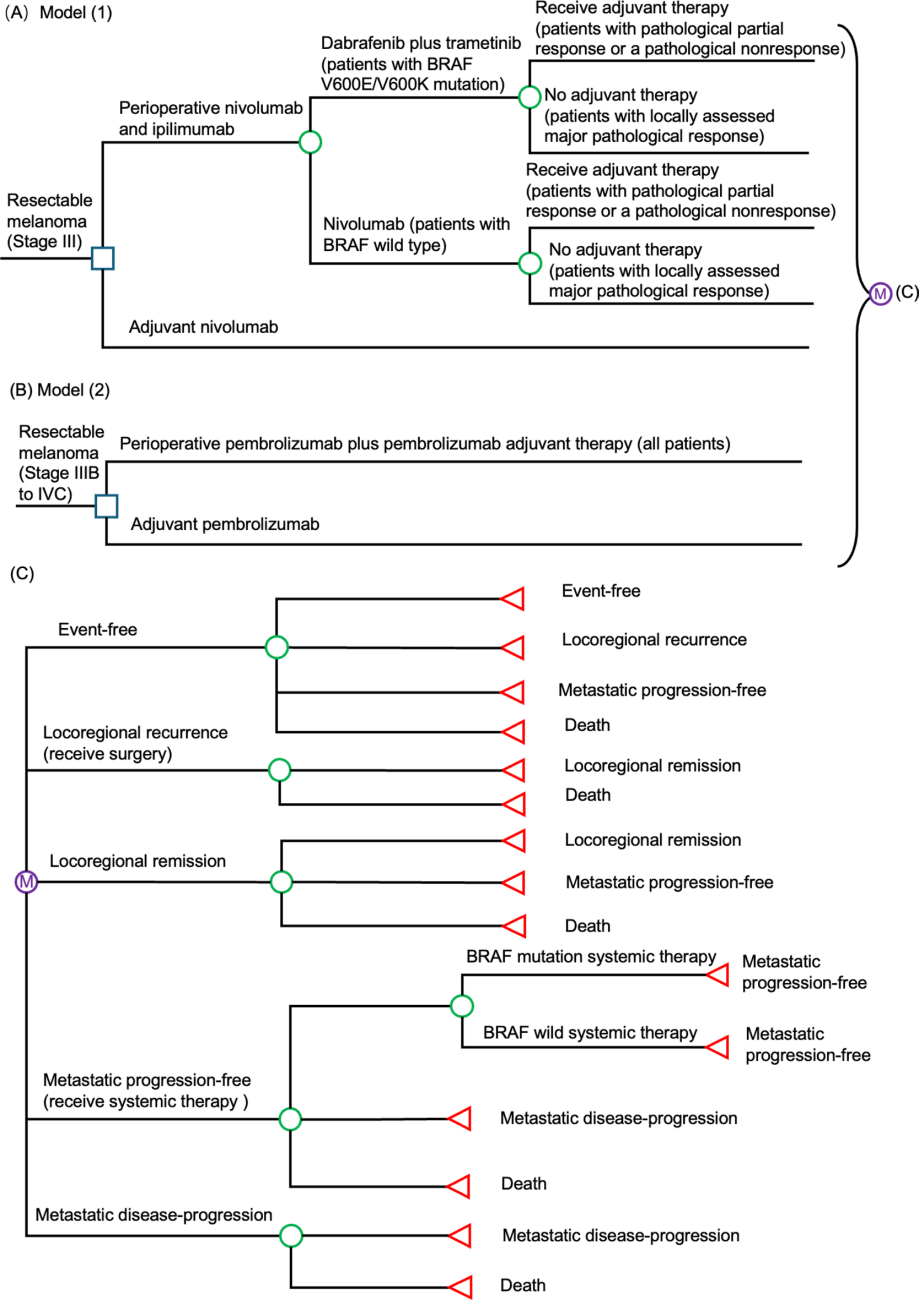
Simplified model for each strategy; (**A**) Perioperative nivolumab plus ipilimumab versus adjuvant nivolumab for stage III resectable melanoma; (**B**) Perioperative pembrolizumab versus adjuvant pembrolizumab for stage IIIB to IVC resectable melanoma; (**C**) Markov model structure of resectable melanoma

Two Markov models (Fig. 1) were developed to simulate the clinical and health economic outcomes of two perioperative treatment strategies (nivolumab plus ipilimumab, and pembrolizumab), each versus the current standard of care (adjuvant therapy) in adult patients with (stage III to IVC) resectable melanoma. The study utilized Markov models to simulate patient progression between mutually exclusive health states. Health outcomes and costs were tallied for each cycle, determined by the specified transition probabilities [10]. The median age of patients with stage III to IVC resectable melanoma in the NADINA trial [8] and SWOG S1801 trial [9] ranged 60–63 years. The present models therefore applied 40 years (with weekly cycle) as the time horizon to allow adequate time for estimation of the lifelong outcome measures, including direct medical cost, life-year gained (LYG) and quality-adjusted life year (QALY) gained by each treatment strategy.

Two immunotherapy were each compared with the corresponding standard adjuvant treatment: Model (1) perioperative nivolumab plus ipilimumab versus adjuvant nivolumab for stage III patients; model (2) perioperative pembrolizumab versus adjuvant pembrolizumab for stage IIIB to IVC patients. Model (1) adopted the treatment regimens of the NADINA trial [8]: Neoadjuvant ipilimumab 80 mg plus nivolumab 240 mg every 3 weeks for 2 cycles, followed by a therapeutic lymph-node dissection. After surgery, those who had a pathological partial response or a pathological nonresponse were further treated with one of the two adjuvant therapies: dabrafenib 150 mg twice daily plus trametinib 2 mg once daily for 46 weeks as adjuvant therapy for patients with BRAF V600E/V600K mutation; nivolumab 480 mg every 4 weeks for 11 cycles in those with BRAF wild type melanoma. The adjuvant nivolumab group underwent a therapeutic lymph node dissection in week 0 followed by 12 cycles of adjuvant 480 mg nivolumab every 4 weeks starting between week 6 and 12. In model (2), the perioperative pembrolizumab group adopted regimens of the SWOG S1801 trial [9]: Neoadjuvant pembrolizumab 200 mg every 3 weeks for 3 cycles, followed by a therapeutic lymph-node dissection and post-surgery adjuvant therapy (pembrolizumab 200 mg every 3 weeks for 15 cycles). In the adjuvant pembrolizumab group, patients underwent surgery without prior neoadjuvant therapy. After surgery, patients received adjuvant therapy with pembrolizumab 200 mg every 3 weeks for 18 cycles [9]. 

The key health states in both Markov models were: Event-free, locoregional recurrence, locoregional remission, metastatic progression-free, metastatic disease-progression, and death. All patients entered the model at the health status of event-free, and transited to locoregional recurrence, metastatic progression-free, or all-cause death based on the strategy-specific weekly transition probabilities derived from results of clinical trials. One-time salvage surgery was used for those who entered the locoregional recurrence state, and the patients might achieve locoregional remission or die from all causes. Patients with locoregional remission might remain in remission, advance to metastatic progression-free, or die from all causes. When advancement to metastatic progression-free occurred, patients were managed by systemic therapy based upon the BRAF status [11]. Depending on the outcomes of systemic therapy, patients might transit to metastatic disease-progression or cancer-associated death.

### Clinical inputs

The clinical model inputs are listed in Table 1. Literature search on MEDLINE (up to 2025) was performed using keywords including “resectable”, “stage III melanoma”, “stage IV melanoma”, “advanced melanoma”, “perioperative”, “neoadjuvant”, “adjuvant”, “event-free survival”, “nivolumab”, “pembrolizumab”, and “immunotherapy”. The selection criteria of clinical trials were: (1) Reports in English language; (2) adult patients with resectable melanoma; and (3) event-free survival was reported. Preferred studies were meta-analyses and randomized controlled trials. When multiple randomized trials were the source for a single model input, the base-case value was set as a weighted average. The upper and lower bounds for the sensitivity analysis were established using the highest and lowest values from these trials.

**Table 1:** Model inputs

Parameters	Base-case value	Range for sensitivity analysis	Distribution	Reference
**Clinical inputs**				
Model (1)				
Proportion of patients with BRAF V600E/V600K mutation	0.45	0.39–0.66	Beta	[12]
Proportion of BRAF V600E/V600K mutation patients with pathological partial response or pathological nonresponse	0.53	0.42–0.64	Beta	[13]
Proportion of BRAF wild type patients with pathological partial response or pathological nonresponse	0.48	0.38–0.57	Beta	[13]
Perioperative nivolumab and ipilimumab versus adjuvant nivolumab				
EFS hazard ratio for BRAF V600E/V600K	0.29	0.11–0.79	Beta	[8]
EFS hazard ratio for BRAF wild type	0.35	0.12–1.03	Beta	[8]
Gompertz distribution for EFS				
Adjuvant nivolumab				
BRAF V600E/V600K mutation				
Shape	-0.02525	-0.04224-(-0.00826)	Cholesky decomposition	[8]
Rate	0.02062	0.01343–0.03166		[8]
BRAF wild type				
Shape	-0.01980	-0.03796-(-0.00163)	Cholesky decomposition	[8]
Rate	0.01373	0.00815–0.02313		[8]
Probability of ADE				
Perioperative nivolumab and ipilimumab				
Diarrhea	3.8%	3.0%-4.6%	Beta	[8]
Rash	3.3%	2.6%-4.0%	Beta	[8]
Adjuvant nivolumab				
Diarrhea	0.6%	0.5%-0.7%	Beta	[8]
Rash	2.4%	0.0%-0.0%	Beta	[8]
Model (2)				
Gompertz distribution for EFS				
Perioperative pembrolizumab				
Shape	-0.04236	-0.06118-(-0.02355)	Cholesky decomposition	[9]
Rate	0.01408	0.00895–0.02215		[9]
Adjuvant pembrolizumab				
Shape	-0.01515	-0.02412-(-0.00618)	Cholesky decomposition	[9]
Rate	0.01392	0.00982–0.01972		[9]
Probability of ADE				
Perioperative pembrolizumab				
Diarrhea	0.7%	0.6%-0.8%	Beta	[9]
Rash	0.9%	0.7%-1.1%	Beta	[9]
Vomiting	0.9%	0.7%-1.1%	Beta	[9]
Adjuvant pembrolizumab				
Diarrhea	0.8%	0.6%-1.0%	Beta	[9]
Rash	3.1%	2.5%-3.7%	Beta	[9]
Vomiting	3.1%	2.5%-3.7%	Beta	[9]
Models (1) and (2)				
Transition probability (weekly)				
From event-free to metastatic progression-free				
Perioperative nivolumab and ipilimumab				
BRAF V600E/V600K mutation	0.0029	0.00261–0.00319	Beta	[8]
BRAF wild type group	0.0023	0.00207–0.00253	Beta	[8]
Adjuvant nivolumab				
BRAF V600E/V600K mutation	0.0106	0.00954–0.01166	Beta	[8]
BRAF wild type group	0.0070	0.00630–0.00770	Beta	[8]
Perioperative pembrolizumab	0.0069	0.00621–0.00759	Beta	[9]
Adjuvant pembrolizumab	0.0068	0.00612–0.00748	Beta	[9]
From locoregional remission to metastatic progression-free	0.01062	0.009558–0.011682	Beta	[17–19]
Death (from event-free)	0.00009	0.000081–0.000099	Beta	[17–19]
Death (locoregional recurrence, or locoregional remission)	0.00078	0.000702–0.000858	Beta	[17–19]
Exponential distribution in patients with metastatic progression-free (rate):				
Progression-free survival for BRAF V600E/V600K	0.00296	0.00269–0.00326	Beta	[22–24]
Overall survival for BRAF V600E/V600K	0.00285	0.00257–0.00317	Beta	[22–24]
Progression-free survival for BRAF wild type	0.00414	0.00376–0.00456	Beta	[22–24]
Overall survival for BRAF wild type	0.00237	0.00214–0.00264	Beta	[22–24]
Hazard ratio (versus pembrolizumab)				
PFS				
Dabrafenib + trametinib	0.46	0.30–0.72	Beta	[22]
Nivolumab + ipilimumab	0.74	0.56–0.98	Beta	[22]
OS				
Dabrafenib + trametinib	1.09	0.68–1.85	Beta	[22]
Nivolumab + ipilimumab	0.79	0.56–1.09	Beta	[22]
Proportional for BRAF V600E/V600K patients				
Dabrafenib + trametinib	68%	Fixed	Dirichlet	[23]
Nivolumab + ipilimumab	19%			[23]
Pembrolizumab	13%			[23]
Proportional for BRAF wild type patients				
Dabrafenib + trametinib	0%	Fixed	Dirichlet	[23]
Nivolumab + ipilimumab	71%			[23]
Pembrolizumab	29%			[23]
**Utility inputs**				
Event-free state	0.9130	0.9040–0.9200	Beta	[26, 27]
Locoregional recurrence state	0.8580	0.833–0.884	Beta	[26, 27]
Locoregional remission	0.8580	0.81–0.846	Beta	[26, 27]
Metastatic progression-free	0.8280	0.551–0.629	Beta	[26, 27]
Metastatic disease-progression	0.5900	0.395–0.561	Beta	[28]
Disutility for ADE				
Diarrhea	-0.047	-0.038-(-0.056)	Beta	[29]
Rash	-0.033	-0.026-(-0.040)	Beta	[29]
Vomiting	-0.048	-0.038-(-0.058)	Beta	[29]
**Cost inputs (USD)**				
Dabrafenib (per mg)	2	2–2	Gamma	[31]
Trametinib (per mg)	378	303–454	Gamma	[31]
Nivolumab (per mg)	32	26–39	Gamma	[32]
Ipilimumab (per mg)	180	144–216	Gamma	[32]
Pembrolizumab (per mg)	58	46–69	Gamma	[32]
T-VEC (per mg)	70	56–84	Gamma	[31]
BRAF testing (per time)	463	370–555	Gamma	[30]
Disease management cost (per week)				
Event-free state	238	191–286	Gamma	[34, 35]
Locoregional recurrence state	261	209–313	Gamma	[34, 36]
Locoregional remission	261	209–313	Gamma	[34, 36]
Metastatic progression-free	1,102	881-1,322	Gamma	[34, 36, 37]
Metastatic disease-progression	1,183	947-1,420	Gamma	[34, 36, 37]
Surgery (per time)	11,107	8,886 − 13,329	Gamma	[34]
Systemic therapy (per patient) for BRAF V600E/V600K patients[a]	556,052	444,842 − 667,262	Gamma	[23, 34]
Systemic therapy (per patient) for BRAF wild type patients[a]	378,411	302,729 − 454,093	Gamma	[23, 34]
Costs for ADE				
Diarrhea	8,676	6,941 − 10,411	Gamma	[38]
Rash	3,178	2,542-3,814	Gamma	[38]
Vomiting	5,294	4,235-6,353	Gamma	[38]

T-VEC: talimogene laherparepvec; ADE: adverse drug event; a: Systemic therapy included pembrolizumab, nivolumab, ipilimumab, nivolumab plus ipilimumab, dabrafenib plus trametinib, and encorafenib plus binimetinib. Details of systemic therapy cost estimation for BRAF V600E/V600K patients and BRAF wild type patients are shown in the Supplementary Materials

In model (1), the proportion of V600E/V600K mutation patients with melanoma was approximated from the findings of a US retrospective cohort study (*n* = 1112) on patients with melanoma [12]. The proportions of pathological partial response and pathological nonresponse for V600E/V600K mutation patients and BRAF wild type patients were approximated from a pooled analysis by the International Neoadjuvant Melanoma Consortium (INMC) which included six clinical trials of anti-PD-1-based immunotherapy or BRAF/MEK targeted therapy [13]. 

The EFS for each treatment strategy were estimated from the Kaplan-Meier event-free survival curves reported by the NADINA trial for model (1), and SWOG S1801 trial for model (2) [8, 9]. For both models, survival data points were extracted from the survival curves to generate patient-level data [14]. The EFS of all four immunotherapy strategies were extrapolated using Gompertz distribution (as the most appropriate distribution based upon the Bayesian information criterion, a visual inspection of fit, and the cure assumption) [15, 16]. Transition probabilities from the event-free to metastatic progression-free were estimated from reported findings of distant metastasis free survival [8, 9]. The transition from locoregional remission to metastatic progression-free was estimated from findings of the KEYNOTE-054 trial [17–19]. The reported survival of systemic therapy in BRAF-mutated and wild-type patients were not significantly different [20]. Exponential rates for progression-free survival and overall survival of patients with metastatic progression-free treated by systemic pembrolizumab therapy were first estimated from individual patient data from the KEYNOTE-006 trial [21]. Relative effects of nivolumab plus ipilimumab and dabrafenib plus trametinib adopted the HRs of corresponding regimens (versus pembrolizumab) reported by a network meta-analysis for both models [22]. Exponential rates for progression free survival and overall survival for patients with BRAF V600E or V600K mutations and for BRAF wild type patients were then estimated by applying HR from a network meta-analysis to survival rate derived from the KEYNOTE-006 trial and weighting these estimates according to the distribution of systemic therapies reported for each group [23]. Probabilities of adverse drug events (including diarrhea, rash and vomiting) were obtained from NADINA trial [8] (for model 1) and SWOG S1801 trial [9] (for model 2).

All-cause death in event-free, locoregional recurrence, and locoregional remission were approximated using the exponential rate of recurrence-free to death from the KEYNOTE-054 trial [17–19]. The cancer-associated mortality (as weekly probability of death) from metastatic progression-free and disease progression was derived from overall survival of patients receiving systemic therapy as estimated using the weighted and hazard adjusted survival curves [22–24]. Background mortality was incorporated by applying age-specific all-cause mortality rates from the United States population, derived from life table data [25]. 

### Utility inputs

The utility inputs are shown in Table 1. The duration of patient-time spent in each health state and the corresponding utility (or disutility) value were used to determine QALY gained. The health state utilities of event-free, locoregional recurrence, locoregional remission, metastatic progression-free, and metastatic disease-progression were obtained from health-related quality-of-life and health economic evaluations of resected stage III melanoma in the US [26, 27]. The utility of metastatic disease-progression state was sourced from a cross-sectional population-based study on preference value for advanced melanoma health states [28]. Disutility values of adverse drug event (including diarrhea, rash and vomiting) were obtained from a health utility study using standard gamble method to elicit different grade III-IV toxicities commonly associated with chemotherapy treatments [29]. The cumulative QALY was discounted annually at a rate of 3%.

### Costs inputs

The cost analysis was conducted on direct medical costs from the perspective of the US payer. Direct costs (Table 1) included drug costs (dabrafenib, trametinib, nivolumab, ipilimumab, pembrolizumab, T-VEC), surgery, BRAF testing, systemic therapy for metastatic progression-free in BRAF V600E/V600K mutation patients and BRAF wild type patients, disease management and adverse event treatment in each health state. The cost of BRAF testing was retrieved from Centers for Medicare & Medicaid Services (CMS) Clinical Laboratory Fee Schedule 2024 [30]. The drug costs of dabrafenib, trametinib and T-VEC were estimated from Medicare Part D Monthly Prescription Drug Plan Formulary and Pharmacy Network Information [31]. The drug costs of nivolumab, ipilimumab, pembrolizumab were estimated from the Medicare Part B Drug Average Sales Price data [32]. The cost of surgery was estimated from the CMS Hospital Outpatient PPS (2022) [33]. The costs of systemic therapy for metastatic progression free disease were estimated separately for patients with BRAF V600E or V600K mutations and for BRAF wild type patients, reflecting differences in available treatment options between the two groups. For BRAF mutated patients, systemic therapy options included pembrolizumab, nivolumab plus ipilimumab, and dabrafenib plus trametinib, whereas BRAF wild type patients were assumed to receive pembrolizumab or nivolumab plus ipilimumab regimens. Treatment specific costs were obtained from US-based cost-effectiveness analyses of metastatic melanoma and combined using weighted averages according to the distribution of systemic therapies reported for each group [23, 34]. Details of the estimation of systemic therapy costs for metastatic disease are showed in Supplementary Materials. Disease management costs for event-free, locoregional recurrence, locoregional remission, metastatic progression-free, and metastatic disease-progression states were derived from the US-based cost-effectiveness analyses and retrospective healthcare database studies [34–37]. The management costs of prominent symptomatic adverse events (including diarrhea, rash and vomiting) were derived from an economic burden study of AEs in cancer care [38]. All cost inputs were converted to year 2025 US dollars using the CCEMG–EPPI Centre Cost Converter v.1.7 [39]. The cumulative costs were discounted with an annual rate of 3%.

### Model validation

Internal validation was conducted by comparing modeled EFS for each treatment arm with the Kaplan Meier curves observed in clinical trials. For the comparison of perioperative nivolumab plus ipilimumab versus adjuvant nivolumab in model (1), the EFS Kaplan Meier curves were derived from the NADINA trial [8]. For the comparison of perioperative pembrolizumab versus adjuvant pembrolizumab in model (2), the EFS Kaplan Meier curves were obtained from the SWOG S1801 trial [9]. The results of the internal validation were presented in the Supplementary Materials Figure S1A and Figure S1B. External validation was performed by comparing modeled overall survival outcomes for all arms with predictions reported in three published and validated economic evaluation models [19, 27, 34]. The results of the external validation were presented in Supplementary Materials Figure S2.

### Cost-effectiveness and sensitivity analyses

The analyses were performed using TreeAge Pro 2025 (TreeAge Software Inc., Williamstown, MA) and Microsoft Excel 16.9 (Microsoft Corporation, Redmond, WA, USA). A treatment option was considered dominated if it resulted in lower QALY at a higher cost versus an alternative, and the dominated option was excluded from further cost-effectiveness evaluation. When a treatment option yielded higher QALY at an increased cost versus an alternative, the incremental cost-effectiveness ratio (ICER) of the more effective option was calculated by the equation: Δcost/ΔQALY. The present analysis adopted 100,000 USD/QALY as the willingness-to-pay (WTP) threshold in the base-case analysis. A treatment option was cost-effective if (1) it gained higher QALY with cost-saving, or (2) it gained higher QALY at increased cost and the ICER was less than the WTP threshold.

A one-way sensitivity analysis was performed on all model inputs to determine the influence of each parameter and to identify critical threshold values. All the parameters were varied over the upper and lower limits, if available. Otherwise, a 95% confidence interval or a range of variation by ± 20% of the base-case value was used. The probabilistic sensitivity analysis was performed using Monte Carlo simulation. The direct cost and QALY of each study arm were recalculated 10,000 times by randomly drawing each of the model inputs from the probability distribution specified in Table 1. The incremental costs and incremental QALY of the 10,000 simulations were presented in scatter plots, and probability to be the cost-effective alternative was evaluated in acceptability curves over a wide range of WTP from 0 to 150,000 USD/QALY.

## Results

### Base-case analysis

Expected total direct medical cost (with breakdown costs in perioperative treatment, before recurrence and after recurrence), QALY, LYG, incremental cost and incremental QALY are shown in Table 2. In model (1), perioperative nivolumab plus ipilimumab (versus adjuvant nivolumab) gained higher QALY (by 2.73) with cost-saving (by USD109,157), and were accepted as cost-effective. Compared with adjuvant nivolumab, the breakdown cost of perioperative treatment and cost before recurrence were higher, while the cost after recurrence was lower in the perioperative nivolumab plus ipilimumab arm.

**Table 2:** Base-case analysis of direct medical cost, QALY and LYG, increment cost, incremental QALY and incremental cost per QALY gained (ICER)

Strategy	Total Cost (USD)	Cost in perioperative treatment (USD)			Cost before recurrence (USD)	Cost after recurrence (USD)	Total QALY	Total LYG	Incremental cost (USD)	Incremental QALY	ICERs (USD per QALY)
		neoadjuvant treatment	surgery	adjuvant treatment							
**Stage III resectable melanoma (model 1)**											
Adjuvant nivolumab	421,681	-	6,364	149,504	6,145	259,668	8.90	10.35	-	-	-
Perioperative nivolumab and ipilimumab	312,525	45,557	6,214	138,733	9,737	112,284	11.62	13.00	-109,157	2.73	Cost-saving
**Stage IIIB to IVC resectable melanoma (model 2)**											
Adjuvant pembrolizumab	459,633	-	6,384	161,260	6,129	285,860	7.90	9.33	-	-	-
Perioperative pembrolizumab	329,476	34,346	5,828	129,697	18,998	140,607	10.19	11.49	-130,157	2.29	Cost-saving

LYG: life-year gained; QALY: quality-adjusted life-year

In model (2), perioperative pembrolizumab (versus adjuvant pembrolizumab) gained higher QALY (by 2.29) with cost-saving (by USD130,157), and were accepted as cost-effective. The breakdown cost of perioperative treatment and cost before recurrence were higher, whilst the cost after disease recurrence were lower in the perioperative pembrolizumab group.

### Sensitivity analysis

**Fig. 2:**
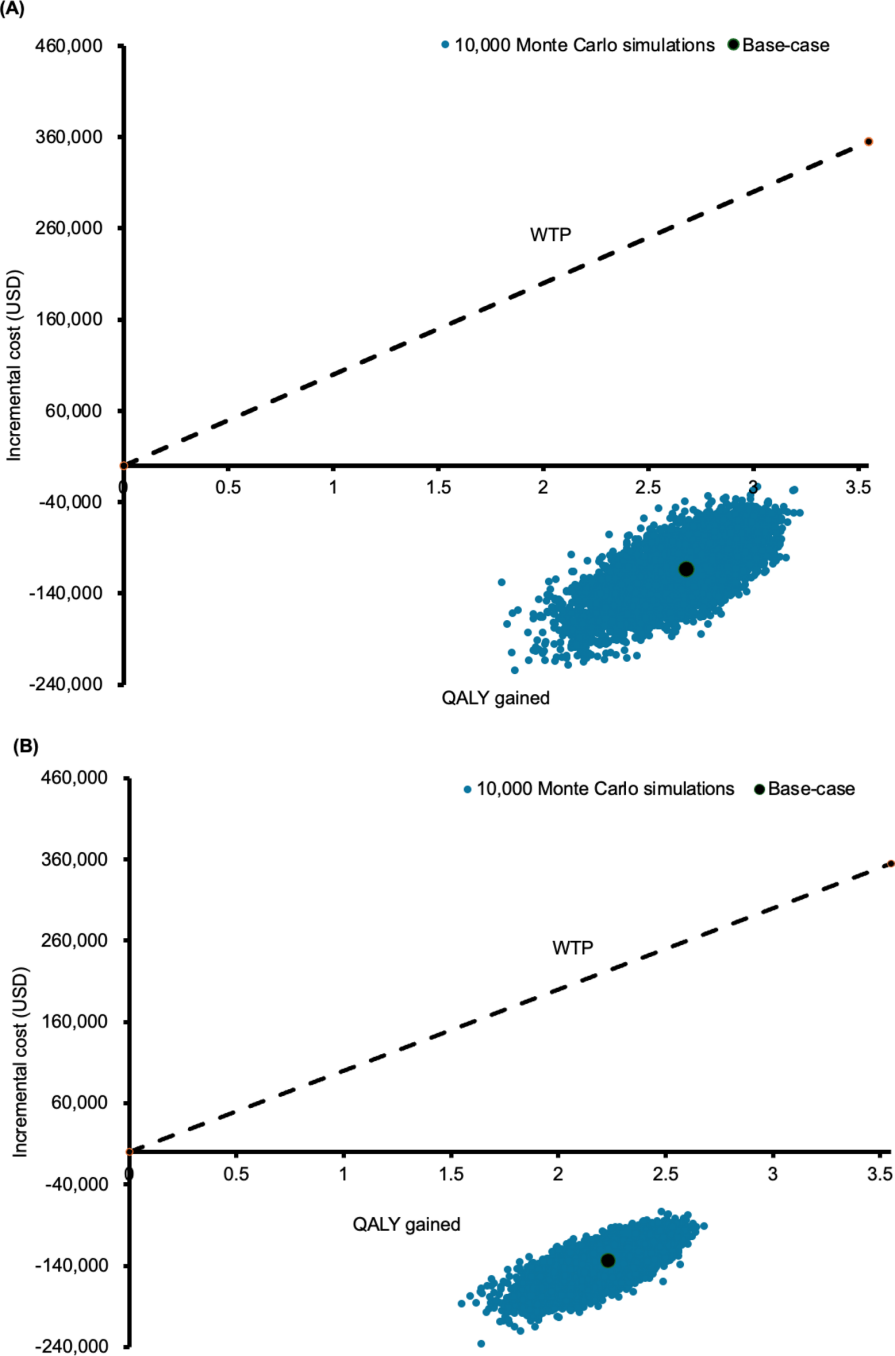
Scatter plots of incremental cost against QALY gained by (**A**) perioperative nivolumab plus ipilimumab versus adjuvant nivolumab; (**B**) perioperative pembrolizumab versus adjuvant pembrolizumab; QALY: quality-adjusted life-year; WTP: willingness-to-pay = 100,000 USD/QALY

One-way sensitivity analysis found that the base-case results were robust to variation of all model inputs at the WTP threshold 100,000 USD/QALY. The probabilistic sensitivity analysis was performed by 10,000 Monte Carlo simulations. In model (1), the perioperative nivolumab plus ipilimumab (versus adjuvant nivolumab) gained additional 2.6610 QALY (95% CI 2.6571–2.6650 *p* < 0.01) with a cost-saving of USD 114,826 (95% CI 114,268 − 115,385 *p* < 0.01). The perioperative nivolumab plus ipilimumab was accepted as cost-effective in 100% of the 10,000 simulations (Fig. 2A). In model (2), the perioperative pembrolizumab (versus adjuvant pembrolizumab) gained additional 2.2461 QALY (95% CI 2.2430–2.2492 *p* < 0.01) with a cost-saving of USD 133,625 (95% CI 133,220 − 134,030 *p* < 0.01). The perioperative pembrolizumab was accepted as cost-effective in 100% of the 10,000 simulations (Fig. 2B).

**Fig. 3:**
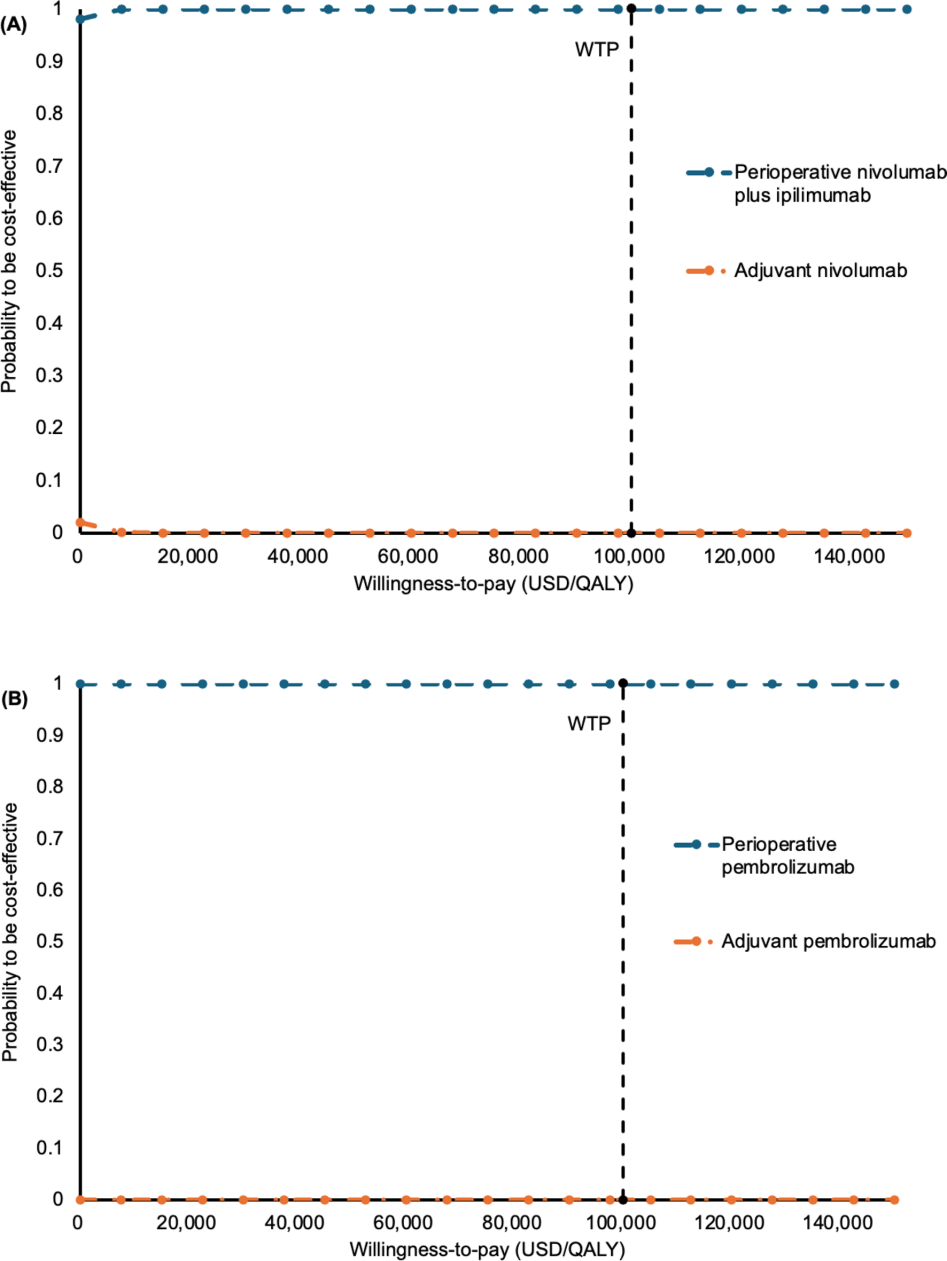
Acceptability curves of (**A**) perioperative nivolumab plus ipilimumab versus adjuvant nivolumab; (**B**) perioperative pembrolizumab versus adjuvant pembrolizumab to be cost-effective against willingness-to-pay; QALY: quality-adjusted life-years; WTP: willingness-to-pay

To examine the acceptability of perioperative nivolumab plus ipilimumab versus adjuvant nivolumab (model 1) and perioperative pembrolizumab versus adjuvant pembrolizumab (model 2), the probabilities of each perioperative strategy to be accepted as cost-effective were shown in the acceptability curves over a wide range of WTP (0–150,000 USD/QALY) (Fig. 3A-B).

## Discussion

The present cost-effectiveness analysis evaluated two perioperative immunotherapy strategies (each versus adjuvant immunotherapy strategies) for patients with resectable melanoma. Our findings showed perioperative nivolumab plus ipilimumab and perioperative pembrolizumab to gain higher QALY with cost-savings when compared to adjuvant nivolumab and adjuvant pembrolizumab, respectively. Breakdown costs showed that the lower total costs observed in the perioperative nivolumab plus ipilimumab and perioperative pembrolizumab groups were primarily driven by lowering the cost after recurrence. Improved event free survival led to reduced use of subsequent systemic therapies for recurrence. The cost-effectiveness of perioperative nivolumab plus ipilimumab was further enhanced by the response-adapted treatment approach, whereby patients who had achieved pathological complete response did not proceed to receive adjuvant therapy, thus resulted in reduced adjuvant treatment cost. One-way sensitivity analysis found that the base-case results were robust to variation of all model inputs for both perioperative nivolumab plus ipilimumab versus adjuvant nivolumab (model 1) and perioperative pembrolizumab versus adjuvant pembrolizumab (model 2). The probabilistic sensitivity analysis supported the base-case findings to be highly robust that the perioperative nivolumab plus ipilimumab (versus adjuvant nivolumab) and perioperative pembrolizumab (versus adjuvant pembrolizumab) were cost-effective in 100% of times at WTP of 100,000 USD/QALY.

A prior cost-effectiveness study found that pembrolizumab (as adjuvant therapy) was cost-effective for resected stage IIB or IIC melanoma when compared with (observation without adjuvant therapy) in the United States [34]. Another US study evaluated the cost-effectiveness of adjuvant pembrolizumab relative to observation alone following complete resection of high-risk stage III melanoma [27]. The adjuvant pembrolizumab was found to be highly cost-effective versus observation alone. A cost-effectiveness analysis evaluated the cost-effectiveness of pembrolizumab versus other adjuvant treatment strategies (routine observation or ipilimumab in the overall population; dabrafenib + trametinib in the BRAF subgroup) for resected high-risk stage III melanoma in the US, and reported that pembrolizumab was cost-effective [40]. Our findings showed that both perioperative nivolumab plus ipilimumab (versus adjuvant nivolumab) and perioperative pembrolizumab (versus adjuvant pembrolizumab) were accepted as cost-effective, consistent with findings of the prior studies.

Perioperative nivolumab plus ipilimumab strategy avoided overtreatment with adjuvant therapy (thus reducing costs), by using pathological response to determine the need for adjuvant therapy, and V600E/V600K mutation status to guide the use of targeted adjuvant therapy [8, 9]. Pathological response is a strong surrogate marker for long-term benefit, as demonstrated by clinical trials included in the pooled analysis by the INMC [13]. In addition, the OpACIN-neo trial demonstrated that neoadjuvant nivolumab plus ipilimumab provided durable survival benefits without the need for subsequent adjuvant systemic therapy [41, 42]. The PRADO trial also supported the omission of adjuvant therapy in patients achieving a major pathological response without compromising outcomes [43]. Furthermore, in the neoadjuvant nivolumab plus ipilimumab group, patients with V600E/V600K mutations received targeted therapy. Real-world studies have suggested that adjuvant targeted therapy may be associated with better outcomes (comparing to immunotherapy) in patients with V600E/V600K mutated melanoma [44]. Further research is warranted to investigate the role of prognostic markers for guiding adjuvant treatment selection and optimizing long-term outcomes in the future. In model (2), perioperative pembrolizumab was cost-saving versus adjuvant pembrolizumab, primarily due to improved event free survival, thus lower the cost after recurrence. Although response-adapted discontinuation of adjuvant therapy following neoadjuvant pembrolizumab was not evaluated in the SWOG S1801 trial [9], such approach may be considered in clinical practice. Incorporating response-adapted strategies for perioperative pembrolizumab is likely to further improve its cost effectiveness and should be explored in future studies.

This study has several limitations. The present study conducted cost-analysis from a payer’s perspective, indirect costs related to productivity loss or caregiver burden were not considered, and might have underestimated the benefits of the perioperative immunotherapy. The current analysis was limited to within trial comparisons (as network meta-analysis was not conducted due to lacking common comparator). The clinical model inputs were extracted from clinical trials, and the heterogeneity of patient characteristics might affect the generalizability of the model results to clinical practice. Rigorous sensitivity analysis on all model inputs was therefore conducted to examine the robustness of model results, and no influential factor was identified.

## Conclusion

Perioperative nivolumab plus ipilimumab and perioperative pembrolizumab appear to gain additional QALY at lower cost when compared with adjuvant immunotherapies for patients with stage III and stage IIIB to IVC resectable melanoma, respectively, from the US payer perspective.

## Supplementary Information

Below is the link to the electronic supplementary material.

Supplementary Material 1

## Data Availability

Data is provided within the manuscript and supplementary files.

## References

[1] Rutkowski P, Mandala M. Perioperative therapy of melanoma: Adjuvant or neoadjuvant treatment. Eur J Surg Oncol. 2024;50(3):107969. 10.1016/j.ejso.2024.107969. 38342039 10.1016/j.ejso.2024.107969

[2] Savage DJ, Switzer B, Parikh R, et al. Patterns in progression from early-stage melanoma to late-stage melanoma: implications for survivorship follow-up. Melanoma Manag. 2024;11(1):2424708. 10.1080/20450885.2024.2424708. 39869444 10.1080/20450885.2024.2424708 PMC11622808

[3] Arnold M, Singh D, Laversanne M, et al. Global burden of cutaneous melanoma in 2020 and projections to 2040. JAMA Dermatology. 2022;158(5):495–503. 10.1001/jamadermatol.2022.0160. 35353115 10.1001/jamadermatol.2022.0160 PMC8968696

[4] Stege H, Haist M, Nikfarjam U, et al. The status of adjuvant and neoadjuvant melanoma therapy, new developments and upcoming challenges. Target Oncol. 2021;16(5):537–52. 10.1007/s11523-021-00840-3. 34554353 10.1007/s11523-021-00840-3 PMC8484171

[5] Alsadiq S, Kartolo A, McWhirter E, Hopman W, Baetz T. Efficacy and safety of adjuvant systemic therapies in trial non-eligible resected stages III and IV melanoma patients. Melanoma Manag. 2025;12(1):2461963. 10.1080/20450885.2025.2461963. 39960333 10.1080/20450885.2025.2461963 PMC11834428

[6] Dimitriou F, Long GV, Menzies AM. Novel adjuvant options for cutaneous melanoma. Ann Oncol. 2021;32(7):854–65. 10.1016/j.annonc.2021.03.198. 33771664 10.1016/j.annonc.2021.03.198

[7] Krishnamoorthy M, Lenehan JG, Maleki Vareki S. Neoadjuvant immunotherapy for high-risk, resectable malignancies: scientific rationale and clinical challenges. JNCI: J Natl Cancer Inst. 2021;113(7):823–32. 10.1093/jnci/djaa216. 33432320 10.1093/jnci/djaa216 PMC8246900

[8] Blank CU, Lucas MW, Scolyer RA, et al. Neoadjuvant nivolumab and ipilimumab in resectable stage III melanoma. N Engl J Med. 2024;391(18):1696–708. 10.1056/NEJMoa2402604. 38828984 10.1056/NEJMoa2402604

[9] Patel SP, Othus M, Chen Y, et al. Neoadjuvant-adjuvant or adjuvant-only pembrolizumab in advanced melanoma. N Engl J Med. 2023;388(9):813–23. 10.1056/NEJMoa2211437. 36856617 10.1056/NEJMoa2211437 PMC10410527

[10] You JHS, Cho WCS, Ming WK, et al. EGFR mutation-guided use of afatinib, erlotinib and gefitinib for advanced non-small-cell lung cancer in Hong Kong - A cost-effectiveness analysis. PLoS ONE. 2021;16(3):e0247860. 10.1371/journal.pone.0247860. 33647045 10.1371/journal.pone.0247860 PMC7920377

[11] Yushak M, Chapman P, Robert C, Kudchadkar R. Systemic therapy options for patients with unresectable melanoma. Am Soc Clin Oncol Educational Book. 2017;37661–72. 10.1200/edbk_174934. 10.1200/EDBK_174934 28561662

[12] Greaves WO, Verma S, Patel KP, et al. Frequency and spectrum of BRAF mutations in a retrospective, single-institution study of 1112 cases of melanoma. J Mol Diagn. 2013;15(2):220–6. 10.1016/j.jmoldx.2012.10.002. 23273605 10.1016/j.jmoldx.2012.10.002 PMC5707183

[13] Menzies AM, Amaria RN, Rozeman EA, et al. Pathological response and survival with neoadjuvant therapy in melanoma: a pooled analysis from the International Neoadjuvant Melanoma Consortium (INMC). Nat Med. 2021;27(2):301–9. 10.1038/s41591-020-01188-3. 33558722 10.1038/s41591-020-01188-3

[14] Guyot P, Ades AE, Ouwens MJNM, Welton NJ. Enhanced secondary analysis of survival data: reconstructing the data from published Kaplan-Meier survival curves. BMC Med Res Methodol. 2012;12(1):9. 10.1186/1471-2288-12-9. 22297116 10.1186/1471-2288-12-9 PMC3313891

[15] Rutherford M, Sweeting M. NICE DSU technical support document 21: flexible methods for survival analysis [Internet]. 2020 [cited 2026 Jan 2]. Available from: http://nicedsu.org.uk/wp-content/uploads/2020/11/NICE-DSU-Flex-Surv-TSD-21_Final_alt_text.pdf.

[16] NICE. NICE DSU technical support document 19. Partitioned survival analysis for decision modelling in health care: A critical review. https://pure.york.ac.uk/portal/en/publications/nice-dsu-technical-support-document-19(4abca204-a8a5-4880-9cce-190049a1daf9).html

[17] Eggermont AMM, Blank CU, Mandala M, et al. Adjuvant pembrolizumab versus placebo in resected stage III melanoma. N Engl J Med. 2018;378(19):1789–801. 10.1056/NEJMoa1802357. 29658430 10.1056/NEJMoa1802357

[18] Eggermont AMM, Blank CU, Mandalà M, et al. Adjuvant pembrolizumab versus placebo in resected stage III melanoma (EORTC 1325-MG/KEYNOTE-054): distant metastasis-free survival results from a double-blind, randomised, controlled, phase 3 trial. Lancet Oncol. 2021;22(5):643–54. 10.1016/s1470-2045(21)00065-6. 33857412 10.1016/S1470-2045(21)00065-6

[19] Lopez-Vinueza C, Urrego-Reyes J, Gutierrez FRS, et al. Cost-effectiveness of pembrolizumab as an adjuvant treatment in colombia for melanoma patients with lymph node involvement after complete resection. Adv Ther. 2023;40(6):2836–54. 10.1007/s12325-023-02484-3. 37129772 10.1007/s12325-023-02484-3 PMC10219874

[20] Zoratti MJ, Devji T, Levine O, Thabane L, Xie F. Network meta-analysis of therapies for previously untreated advanced BRAF-mutated melanoma. Cancer Treat Rev. 2019;74:43–8. 10.1016/j.ctrv.2019.02.001. 30798169 10.1016/j.ctrv.2019.02.001

[21] Robert C, Schachter J, Long GV, et al. Pembrolizumab versus ipilimumab in advanced melanoma. N Engl J Med. 2015;372(26):2521–32. 10.1056/NEJMoa1503093. 25891173 10.1056/NEJMoa1503093

[22] Franken MG, Leeneman B, Gheorghe M, et al. A systematic literature review and network meta-analysis of effectiveness and safety outcomes in advanced melanoma. Eur J Cancer. 2019;123:58–71. 10.1016/j.ejca.2019.08.032. 31670077 10.1016/j.ejca.2019.08.032

[23] Owen CN, Shoushtari AN, Chauhan D, et al. Management of early melanoma recurrence despite adjuvant anti-PD-1 antibody therapy(☆). Ann Oncol. 2020;31(8):1075–82. 10.1016/j.annonc.2020.04.471. 32387454 10.1016/j.annonc.2020.04.471 PMC9211001

[24] Long GV, Carlino MS, McNeil C, et al. Pembrolizumab versus ipilimumab for advanced melanoma: 10-year follow-up of the phase III KEYNOTE-006 study. Ann Oncol. 2024;35(12):1191–9. 10.1016/j.annonc.2024.08.2330. 39306585 10.1016/j.annonc.2024.08.2330

[25] Statistics NCfH. Mortality in the United States. 2023 Dec 16;2024. https://www.cdc.gov/nchs/products/databriefs/db521.htm

[26] Bottomley A, Coens C, Mierzynska J, et al. Adjuvant pembrolizumab versus placebo in resected stage III melanoma (EORTC 1325-MG/KEYNOTE-054): health-related quality-of-life results from a double-blind, randomised, controlled, phase 3 trial. Lancet Oncol. 2021;22(5):655–64. 10.1016/s1470-2045(21)00081-4. 33857414 10.1016/S1470-2045(21)00081-4

[27] Bensimon AG, Zheng-Yi Z, Madeline J, et al. Cost-effectiveness of pembrolizumab for the adjuvant treatment of resected high-risk stage III melanoma in the United States. J Med Econ. 2019;22(10):981–93. 10.1080/13696998.2019.1609485. 31012765 10.1080/13696998.2019.1609485

[28] Beusterien KM, Szabo SM, Kotapati S, et al. Societal preference values for advanced melanoma health states in the United Kingdom and Australia. Br J Cancer. 2009;101(3):387–9. 10.1038/sj.bjc.6605187. 19603025 10.1038/sj.bjc.6605187 PMC2720221

[29] Nafees B, Stafford M, Gavriel S, Bhalla S, Watkins J. Health state utilities for non small cell lung cancer. Health Qual Life Outcomes. 2008;6:84. 10.1186/1477-7525-6-84. 18939982 10.1186/1477-7525-6-84 PMC2579282

[30] 24CLABQ1. Centers for Medicare & Medicaid Services. https://www.cms.gov/medicare/payment/fee-schedules/clinical-laboratory-fee-schedule-clfs/files/24clabq1. Accessed 01/10, 2024.

[31] Monthly Prescription Drug Plan Formulary and Pharmacy Network Information. Centers for Medicare & Medicaid Services. https://data.cms.gov/provider-summary-by-type-of-service/medicare-part-d-prescribers/monthly-prescription-drug-plan-formulary-and-pharmacy-network-information. Accessed 01/10, 2024.

[32] Medicare Part B. Drug average sales price. https://www.cms.gov/medicare/payment/fee-for-service-providers/part-b-drugs/average-drug-sales-price.

[33] Hospital Outpatient CMS. PPS 2022. https://www.cms.gov/medicare/payment/prospective-payment-systems/hospital-outpatient.

[34] Zhang S, Bensimon AG, Xu R, et al. Cost-effectiveness analysis of pembrolizumab as an adjuvant treatment of resected stage IIB or IIC melanoma in the United States. Adv Therapy. 2023;40(7):3038–55. 10.1007/s12325-023-02525-x. 10.1007/s12325-023-02525-x PMC10271902 37191852

[35] CMS Physician Fee Schedule; 2022. https://www.cms.gov/medicare/payment/fee-schedules/physician

[36] Tarhini A, Ghate SR, Ionescu-Ittu R, et al. Postsurgical treatment landscape and economic burden of locoregional and distant recurrence in patients with operable nonmetastatic melanoma. Melanoma Res. 2018;28(6):618–28. 10.1097/cmr.0000000000000507. 30216199 10.1097/CMR.0000000000000507 PMC6221390

[37] Klink AJ, Chmielowski B, Feinberg B, et al. Health care resource utilization and costs in first-line treatments for patients with metastatic melanoma in the United States. J Manag Care Spec Pharm. 2019;25(8):869–77. 10.18553/jmcp.2019.18442. 30945965 10.18553/jmcp.2019.18442 PMC10397699

[38] Patel A, Schuldt R, Sussell J. New estimates of the costs of adverse events in patients with cancer. PLoS ONE. 2025;20(9):e0332703. 10.1371/journal.pone.0332703. 41021622 10.1371/journal.pone.0332703 PMC12478943

[39] CCEMG - EPPI-Centre Cost Converter. https://eppi.ioe.ac.uk/costconversion/. Accessed 01/10, 2024.

[40] Bensimon AG, Zhou ZY, Jenkins M, et al. An Economic evaluation of pembrolizumab versus other adjuvant treatment strategies for resected high-risk stage III melanoma in the USA. Clin Drug Investig. 2020;40(7):629–43. 10.1007/s40261-020-00922-6. 32418051 10.1007/s40261-020-00922-6 PMC7311503

[41] Rozeman EA, Hoefsmit EP, Reijers ILM, et al. Survival and biomarker analyses from the OpACIN-neo and OpACIN neoadjuvant immunotherapy trials in stage III melanoma. Nat Med. 2021;27(2):256–63. 10.1038/s41591-020-01211-7. 33558721 10.1038/s41591-020-01211-7

[42] Reijers ILM, Menzies AM, Lopez-Yurda M, et al. Impact of personalized response-directed surgery and adjuvant therapy on survival after neoadjuvant immunotherapy in stage III melanoma: Comparison of 3-year data from PRADO and OpACIN-neo. Eur J Cancer. 2025;214:115141. 10.1016/j.ejca.2024.115141. 39602990 10.1016/j.ejca.2024.115141

[43] Reijers ILM, Menzies AM, van Akkooi ACJ, et al. Personalized response-directed surgery and adjuvant therapy after neoadjuvant ipilimumab and nivolumab in high-risk stage III melanoma: the PRADO trial. Nat Med. 2022;28(6):1178–88. 10.1038/s41591-022-01851-x. 35661157 10.1038/s41591-022-01851-x

[44] Amaral T, Nanz L, Higuita LMS, et al. A comparison of real-world data on adjuvant treatment in patients with stage III BRAF V600 mutated melanoma – Results of systematic literature research. Eur J Cancer. 2025;215:115160. 10.1016/j.ejca.2024.115160. 39673834 10.1016/j.ejca.2024.115160 PMC7618644

