# Supplementary material for: Perioperative immunotherapy for advanced resectable melanoma: a cost-effectiveness analysis
Source: BMC Health Serv Res. 2026 Feb 26;26:450. doi: 10.1186/s12913-026-14258-y (PMC13040867; doi:10.1186/s12913-026-14258-y)
Supplement: Supplementary file 1 — Supplementary Material 1 [file 12913_2026_14258_MOESM1_ESM.docx]

**Supplementary Materials**

**Estimation of** **systemic therapy costs for metastatic progression free disease**

The per patient cost of systemic therapy for metastatic progression free disease was estimated separately for patients with BRAF V600E or V600K mutations and for BRAF wild type patients.

For patients with BRAF V600E or V600K mutations, three systemic treatment options were considered: Pembrolizumab, nivolumab plus ipilimumab, and dabrafenib plus trametinib. Based on literature reported treatment distributions, 68% of patients were assumed to receive dabrafenib plus trametinib, 19% received nivolumab plus ipilimumab, and 13% received pembrolizumab.^1^ The per patient costs of dabrafenib plus trametinib, nivolumab plus ipilimumab, and pembrolizumab were USD613,892, USD415,058, and USD211,524, respectively, derived from a published US cost effectiveness analysis of advanced melanoma treatments.^2^ The weighted average cost per patient was calculated:

0.68 × USD613,892 + 0.19 × USD415,058 + 0.13 × USD211,524 = USD524,451 per patient (in year 2023 USD). This value was further converted to year 2025 USD (using the CCEMG–EPPI Centre Cost Converter (version 1.7)): USD556,052 per patient.

For patients with BRAF wild type melanoma, two systemic treatment options were considered: Pembrolizumab, and nivolumab plus ipilimumab. According to literature reported treatment distributions, 71% of patients received nivolumab plus ipilimumab and 29% received pembrolizumab, with no use of BRAF targeted therapy in this group.^1^ Using the costs per patient costs for nivolumab plus ipilimumab (USD415,058) and pembrolizumab (USD211,524), the weighted average cost per patient was calculated:

0.71 × USD415,058 + 0.29 × USD211,524 = USD356,905 per patient (in year 2023 USD). This value was further converted to year 2025 USD (using the CCEMG–EPPI Centre Cost Converter (version 1.7)): USD378,411 per patient.

Reference:

1. Owen CN, Shoushtari AN, Chauhan D, et al. Management of early melanoma recurrence despite adjuvant anti-PD-1 antibody therapy(☆). *Ann Oncol*. 2020;31(8):1075-1082. doi:10.1016/j.annonc.2020.04.471

2. Zhang S, Bensimon AG, Xu R, et al. Cost-Effectiveness Analysis of Pembrolizumab as an Adjuvant Treatment of Resected Stage IIB or IIC Melanoma in the United States. *Advances in Therapy*. 2023;40(7):3038-3055. doi:10.1007/s12325-023-02525-x


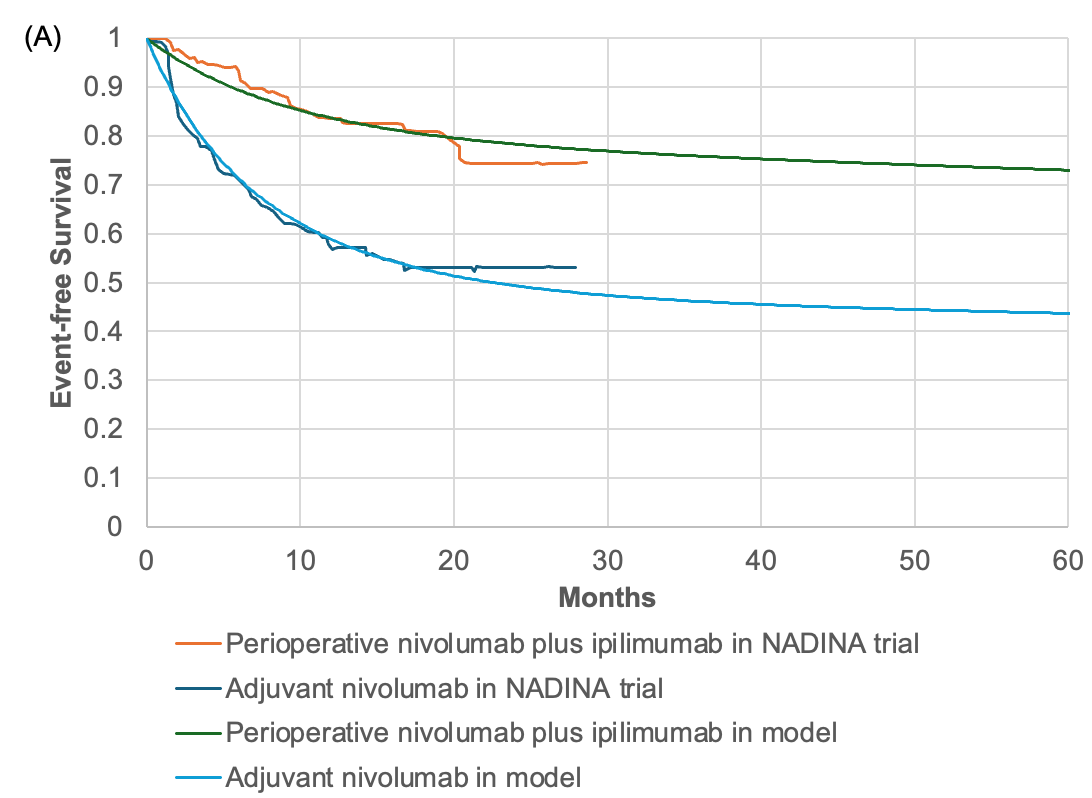

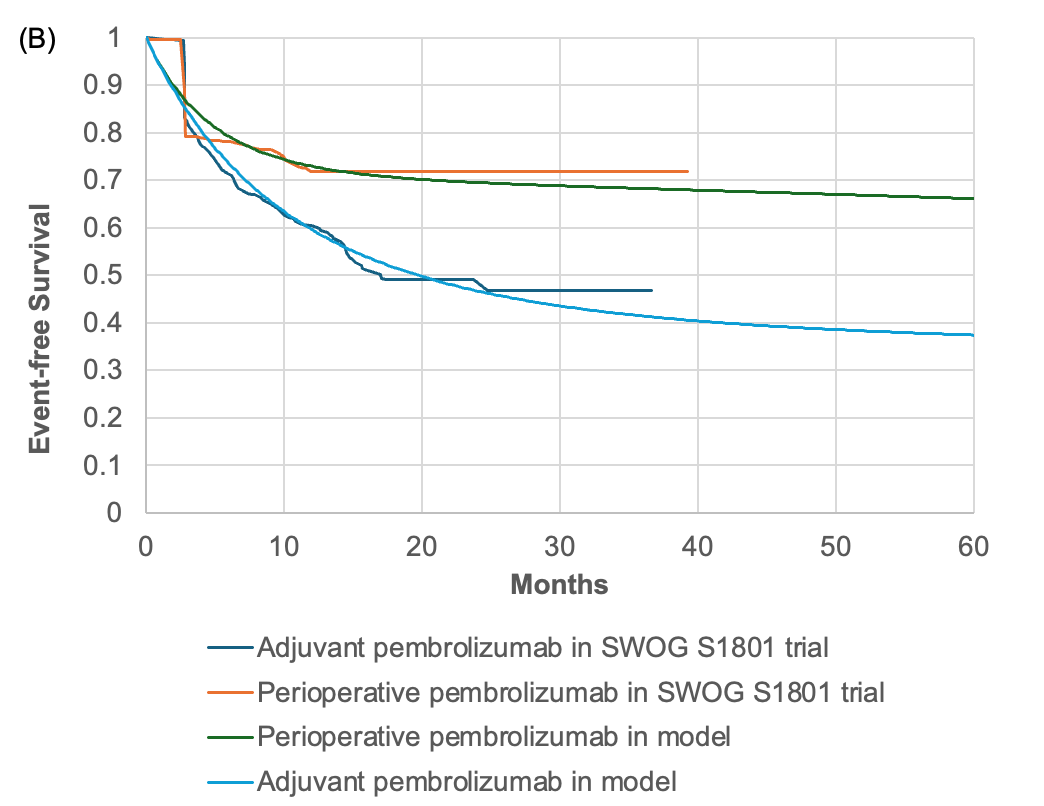


**Figure S1 Internal validation of (A): perioperative nivolumab plus ipilimumab versus adjuvant nivolumab; (B) perioperative pembrolizumab versus adjuvant pembrolizumab**


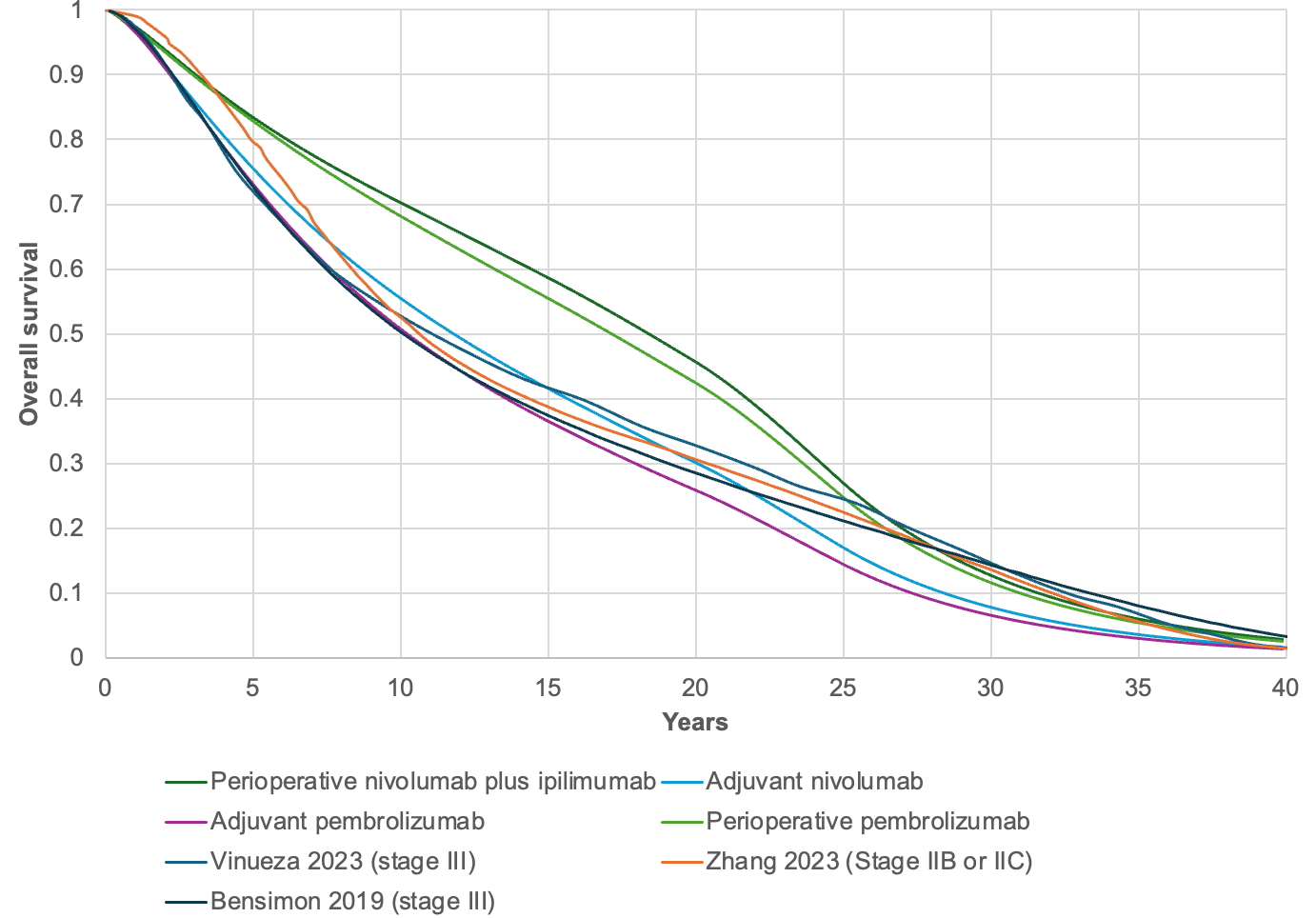


**Figure S2 External validation of perioperative nivolumab plus ipilimumab, adjuvant nivolumab, perioperative pembrolizumab and adjuvant pembrolizumab compared with the overall survival published in previous economic evaluations**
